# Sensation Seeking, Deviant Peer Affiliation, and Internet Gaming Addiction Among Chinese Adolescents: The Moderating Effect of Parental Knowledge

**DOI:** 10.3389/fpsyg.2018.02727

**Published:** 2019-01-11

**Authors:** Yunlong Tian, Chengfu Yu, Shuang Lin, Junming Lu, Yi Liu, Wei Zhang

**Affiliations:** ^1^School of Education, Guangzhou University, Guangzhou, China; ^2^School of Education, Center for Brain and Cognitive Sciences, Guangzhou University, Guangzhou, China; ^3^Faculty of Social and Public Administration, Guangdong Baiyun University, Guangzhou, China; ^4^School of Psychology, South China Normal University, Guangzhou, China

**Keywords:** Internet gaming addiction (IGA), sensation seeking, deviant peer affiliation, parental knowledge, adolescence

## Abstract

Although there is abundant evidence that an association between sensation seeking and adolescent Internet gaming addiction (IGA) exists, research has provided little insight into why adolescents with high sensation seeking are more likely to be focused on Internet and video games. Grounded in the social development model and ecological systems theory, this study investigated whether deviant peer affiliation mediated the relationship between sensation seeking and adolescent IGA, and whether this indirect link was moderated by parental knowledge. Participants were 1293 Chinese adolescents (49.65% male, *M*_age_ = 12.89 ± 0.52 years) who completed questionnaires assessing sensation seeking, deviant peer affiliation, parental knowledge, and IGA. Structural equation models revealed that the positive association between sensation seeking and adolescent IGA was partially mediated by deviant peer affiliation. In addition, this indirect link was significantly moderated by parental knowledge. Specifically, the indirect path from sensation seeking to adolescent IGA was stronger for adolescents with low parental knowledge than for those with high parental knowledge. Identifying the role of peers and parents in the onset of adolescent IGA has key implications for prevention and intervention.

## Introduction

Internet gaming addiction (IGA) refers to an uncontrollable, excessive, and compulsive use of Internet games (Block, 2008). Considerable evidence has indicated that IGA contributes to challenges with meeting daily responsibilities, engaging in social and leisure pursuits, having a healthy lifestyle, and may lead to an increased risk for psychopathology (King and Delfabbro, 2016; Musetti et al., 2016; Hu et al., 2017). Moreover, research has indicated that Chinese adolescents have higher IGA rates than their peers in the United States and Europe (Block, 2008; Yu et al., 2015). Thus, identifying the mechanisms of contributing to the development of adolescent IGA is essential for the establishment of scientific prevention and intervention programs.

Sensation seeking has been significantly positively related to problem behaviors, such as substance use and IGA (Mehroof and Griffiths, 2010; Drane et al., 2017; Hu et al., 2017). For example, Mehroof and Griffiths (2010) found that sensation seeking was positively related to IGA in university students in the United Kingdom. Similarly, Hu et al. (2017) reported that sensation seeking significantly and positively predict IGA in Chinese adolescents. These findings highlight the positive association between sensation seeking and adolescent IGA. However, previous research has focused primarily on the direct association between sensation seeking and adolescent IGA; thus, the mediating mechanism underlying this relationship remains largely unknown. In this study that was grounded in the social development model (Hawkins and Weis, 1985) and ecological systems theory (Bronfenbrenner, 2005), we aimed to investigate whether deviant peer affiliation mediated the relationship between sensation seeking and adolescent IGA, and whether this indirect link was moderated by parental knowledge.

According to the social development model (Hawkins and Weis, 1985), sensation seeking may increase the risk of adolescents affiliating with deviant peers, which in turn may increase adolescent problem behavior such as IGA. In the process of adolescents’ social development, sensation seeking influences one’s choice of peers, and the characteristics of one’s peer group, in turn, may contribute to IGA. Previous investigations have found that adolescents with high sensation seeking are more likely to report having high levels of deviant peer affiliation (Hampson et al., 2008), and may positively predict their deviant peer affiliation (Yanovitzky, 2005; Hampson et al., 2008). Further, deviant peer affiliation has been shown to mediate the association between sensation seeking and problem behaviors (Mann et al., 2015). Additionally, adolescent engagement in various socially undesirable behaviors with their deviant peers is a primary risk factor for the development of Internet addiction (Li et al., 2013).

Fergusson et al. (2002) has noted a possible mechanism between deviant peer affiliation and the risk of adolescent problem behaviors, such as delinquency and alcohol abuse. In this process, a deviant peer may create a social milieu that prompts an adolescent into participating in deviant activities leading to problem behaviors. This peer environment may reinforce adolescents’ problem behaviors through imitation, social learning, and social facilitation (Fergusson et al., 2002). Other researchers have suggested that a homogeneous process of selecting peers increases the possibility of problem behaviors (Mann et al., 2015). Joining a deviant peer group is one way for adolescents with high sensation seeking to express themselves, as youth engaging in deviant behavior typically have a greater need for sensation seeking than do their typically developing peers (Caspi et al., 2005; Hampson et al., 2008). Given the research that has established the relationship between deviate peers and sensation seeking, and the relationship between sensation seeking and IGA, we proposed the following as our first hypothesis, deviant peer affiliation would mediate the relationship between sensation seeking and adolescent IGA.

Although sensation seeking is generally believed to be associated with IGA, but not all adolescents are equally influenced by sensation seeking. According to the ecological systems theory (Bronfenbrenner, 2005), the risk of adolescent IGA is determined by the interaction of individual factors (e.g., sensation seeking) and environment factors (e.g., parental knowledge, deviant peer affiliation). On the one hand, empirical studies have shown the interactive roles of parental knowledge and sensation seeking in adolescent development (Thompson et al., 2015; Su et al., 2017; Rioux et al., 2018). Parental knowledge has been found to moderate the relationship between sensation seeking and adolescent substance use (Rioux et al., 2018). Specifically, the positive relationship between sensation seeking and adolescent substance use was stronger for adolescents with low parental knowledge than for those with high parental knowledge. In summary, among adolescents with high level parental knowledge, parents may be more likely to impose and enforce a set of rules about where their children can go and whom they can spend their time with, which in turn, would reduce their risk of problem behavior, including IGA, deviant peer affiliation.

**FIGURE 1:**
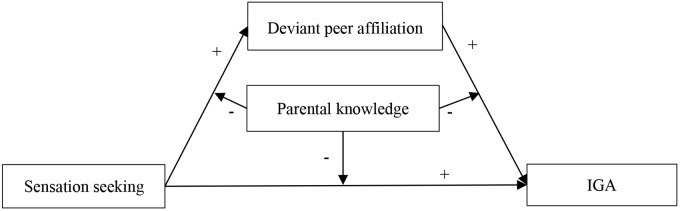
The conceptual model of the proposed moderated mediation framework. IGA, Internet gaming addiction.

On the other hand, although peers are at the center of the social development of adolescents, parents still have considerable influence on adolescents concerning interpersonal communication and behavior formation (Grotevant, 1997). Empirical evidence has consistently demonstrated that parental knowledge can buffer the effects of deviant peer affiliation on adolescent development (Jiang et al., 2016; Xu et al., 2017). For example, Jiang et al. (2016) found that parental knowledge had a protective effect on the relationship between children’s deviant peer affiliation and substance use (e.g., alcohol and cigarette). To date, little research has examined the moderating effect of parental knowledge on the direct or indirect pathways from sensation seeking to IGA. Based on the above theoretical analyses and empirical evidence, we developed our second hypothesis that parental knowledge would moderate the indirect relationship between sensation seeking and adolescent IGA. This indirect association would be stronger for adolescents with low levels of parental knowledge and weaker among adolescents with high levels of parental knowledge. Figure 1 illustrates the theoretical model between sensation seeking, adolescent IGA, deviant peer affiliation and parental knowledge.

## Materials and Methods

### Participants

The sample included 1293 participants who were recruited from four junior middle schools in the southern Chinese province of Guangdong. There were 642 boys (49.65%) and 651 girls (50.35%) in the sample. Participants were tested using a random cluster approach and anonymous questionnaires to avoid social desirability biases. The participants ranged in age from 11 to 15 years old (*M*_age_ = 12.89 ± 0.52 years).

### Measures

#### Sensation Seeking

Sensation seeking was assessed by the 6-item Chinese version of the sensation seeking scale (Li et al., 2010). All items were rated on a 6-point scale ranging from 1 (*almost always untrue of you*) to 6 (*almost always true of you*). The mean of all 6 items was calculated; higher scores reflect greater sensation seeking. A Cronbach’s α of 0.75 was found for the sample in this study.

#### Deviant Peer Affiliation

Deviant peer affiliation was assessed by the 12-item deviant peer affiliation questionnaire (Zhu et al., 2015). This questionnaire asked participants to report how many of their friends expressed deviant behaviors in the past 6 months (e.g., “How many of your friends damaged public property in the past 6 months?” and “How many of your friends stole goods in the past 6 months?”). The questionnaire was answered using a 5-point scale (from 1 = *never* to 5 = *six or more times*). The mean of all 12 items was calculated; higher scores reflected greater deviant peer affiliation. A Cronbach’s α = 0.90 was found in this study.

#### Parental Knowledge

Parental knowledge was assessed by a short form of the parental monitoring questionnaire (Jiang et al., 2016). Respondents were asked to report the level of parental knowledge of their activities during their leisure time, after school, and others. This questionnaire used a 3-point scale (1 = *know little* to 3 = *know much*) to examine the level of parental knowledge in past 6 months. The mean of all 5 items was calculated; higher scores reflect greater levels of parental knowledge. A Cronbach’s α = 0.72 was found in this study.

#### IGA

Internet gaming addiction was assessed with the 11-item IGA questionnaire (Yu et al., 2015). Participants were asked to respond to all items by indicating their level of Internet gaming behavior (e.g., “Have you tried to play online games less often or for shorter periods, but are unsuccessful?”). The questionnaire used a 3-point scale (from 1 = *never* to 3 = *yes*). According to previous research, these answers were coded as *never* = 0, *sometimes* = 0.5, *yes* = 1. The total score of all 11-item was calculated; higher scores reflect greater levels of IGA. The Cronbach’s α was 0.83 in this study.

### Procedure

Ethics approval for this study was obtained from the Certification of Ethics Review Committee of Education School, Guangzhou University. We obtained written consent informs from the school, all participants, their parents, and teachers. An experimenter administered the self-report questionnaires to adolescents in their classrooms. To encourage honest reporting, randomly cluster selected participants were given approximately 30 min to complete the anonymous questionnaires. Participants were also told that they needed to complete the questionnaire by themselves and that the researcher would protect their privacy. The ethics committee of the author’s university approved all questionnaires and procedures.

### Statistical Analyses

SPSS 21.0 was used to analyze data and examine the correlations between the variables. According to a procedure suggested by Muthén and Muthén (1998/2012), we perform structural equation modeling using Mplus 7.1 for data analysis. The method of full-information maximum likelihood was used to examine moderated mediation model of this study. Three conditions were used to assess model fit: the chi-square and normed chi-square (χ^2^/*df*), the comparative fit index (CFI), the root mean square error of approximation (RMSEA). A relatively good fit was defined using the criteria: χ^2^/*df* ≤ 3, CFI ≥ 0.95, and RMSEA ≤ 0.06 (Chou and Bentler, 1995; Kline, 2005). Furthermore, previous research has shown that gender, age, and socioeconomic status of adolescent were relationship with IGA (Yu et al., 2015), so these demographic factors were controlled for in the data analyses.

## Results

### Descriptive Statistics

Means, standard deviations, and correlations are displayed in Table 1. Sensation seeking was positively correlated with deviant peer affiliation and IGA. Deviant peer affiliation and IGA were also positively correlated. Parental knowledge was negatively associated with deviant peer affiliation and IGA.

**Table 1:** Descriptive statistics and correlations for all variables.

Variable	*M*	*SD*	1	2	3	4
(1) Sensation seeking	2.37	0.71	1.00			
(2) Parental knowledge	2.58	0.49	-0.05	1.00		
(3) Deviant peer affiliation	1.42	0.72	0.20^∗∗^	-0.22^∗∗^	1.00	
(4) IGA	1.61	1.75	0.16^∗∗^	-0.26^∗∗^	0.27^∗∗^	1.00


*IGA, Internet gaming addiction. ^∗∗^*p* < 0.01.*

### Testing for Mediation

**FIGURE 2:**
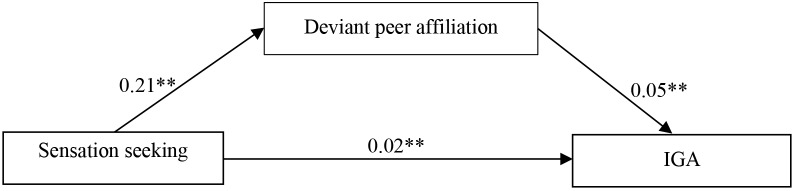
The path model of mediating role of deviant peer affiliation between sensation seeking and IGA. Gender, age, SES were included in model as control variables, but non-significant paths and these variables were not displayed. Following were significant of those paths: SES to deviant peer affiliation (*b* = -0.05, SE = 0.0113, *t* = -4.51, *p* < 0.01) and IGA (*b* = -0.01, SE = 0.0024, *t* = -2.29, *p* < 0.05), gender to IGA (*b* = 0.10, SE = 0.0082, *t* = 11.99, *p* < 0.01). IGA, Internet gaming addiction. ^∗∗^*p* < 0.01.

The hypothesized mediation model included a good fit to the data, χ^2^/*df* = 0.053, CFI = 1.000, RMSEA = 0.000. Figure 2 displays the results of this model. Sensation seeking positively predicted deviant peer affiliation (*b* = 0.21, SE = 0.031, *t* = 6.86, *p* < 0.01) and positively predicted IGA (*b* = 0.02, SE = 0.007, *t* = 12.86, *p* < 0.01), deviant peer affiliation positively predicted IGA (*b* = 0.05, SE = 0.006, *t* = 8.28, *p* < 0.01). Furthermore, bootstrapping analyses identified that deviant peer affiliation partially mediated the pathway from sensation seeking to IGA (indirect effect = 0.010, SE = 0.002, 95% CI = [0.007, 0.015]).

### Testing for Moderated Mediation

**FIGURE 3:**
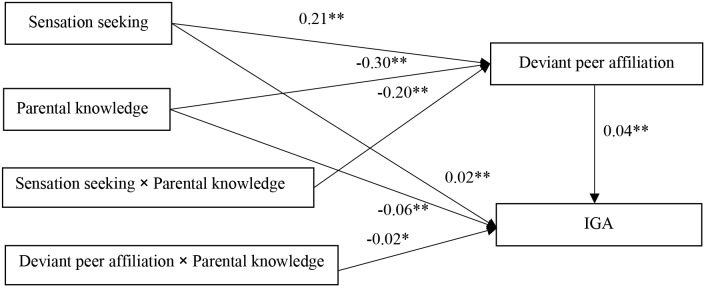
The path model of moderating role of parental knowledge on the indirect link between sensation seeking and IGA of adolescent. Gender, age, SES were included in model as control variables, but non-significant paths and these variables were not displayed. Following were significant of those paths: SES to deviant peer affiliation (*b* = -0.04, SE = 0.0112, *t* = -3.64, *p* < 0.01) and gender to IGA (*b* = 0.10, SE = 0.0080, *t* = 11.68, *p* < 0.01). IGA, Internet gaming addiction. ^∗^*p* < 0.05; ^∗∗^*p* < 0.01.

**FIGURE 4:**
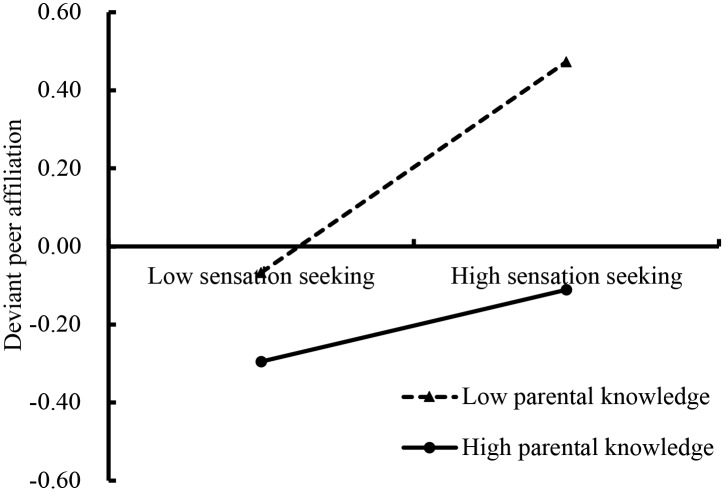
Deviant peer affiliation among adolescents as a function of sensation seeking and parental knowledge.

**FIGURE 5:**
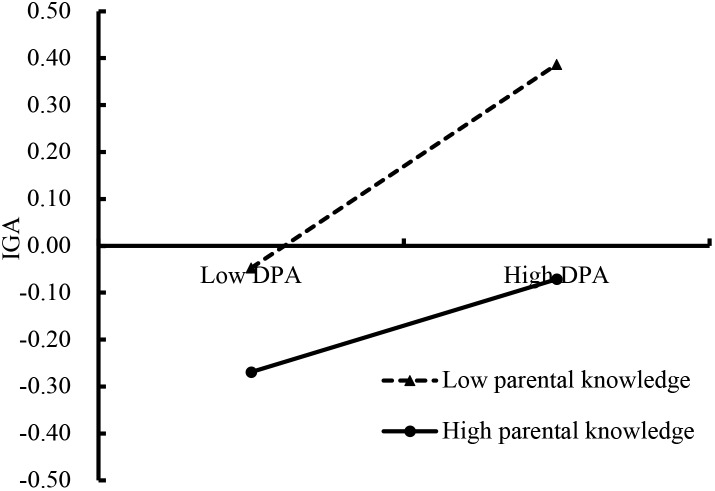
Internet gaming addiction among adolescents as a function of deviant peer affiliation and parental knowledge. DPA, deviant peer affiliation; IGA, Internet gaming addiction.

The moderated mediation model is shown in Figure 3, and was found to have a good fit to the data: χ^2^/*df* = 3.91, CFI = 0.938, RMSEA = 0.047. Sensation seeking negatively predicted IGA (*b* = 0.02, SE = 0.006, *t* = 3.16, *p* < 0.01). Additionally, deviant peer affiliation positively predicted IGA (*b* = 0.04, SE = 0.0061, *t* = 5.83, *p* < 0.01). Parental knowledge moderated the association between sensation seeking and deviant peer affiliation (*b* = -0.20, SE = 0.061, *t* = -3.36, *p* < 0.01). Furthermore, we plotted the predicted sensation seeking increase deviant peer affiliation was much stronger for adolescents with low (*b* = 0.30, SE = 0.043, *t* = 7.14, *p* < 0.01) and high (*b* = 0.12, SE = 0.04, *t* = 3.08, *p* < 0.01) levels of parental knowledge (see Figure 4). Moreover, the interaction between parental knowledge and deviant peer affiliation was significant predict IGA (*b* = -0.02, SE = 0.011, *t* = -2.19, *p* < 0.05). The simple slope testing shown that for low parental knowledge adolescent (*b* = 0.05, SE = 0.007, *t* = 6.86, *p* < 0.01), higher deviant peer affiliation was associated with higher IGA than high parental knowledge adolescent (*b* = 0.03, SE = 0.084, *t* = 3.04, *p* < 0.01). These results are depicted in Figure 5.

The results for the conditional indirect effects were found to be significant for adolescents with lower parental knowledge (indirect effect = 0.014, SE = 0.004, 95% CI [0.008, 0.023]) and for adolescents with higher parental knowledge (indirect effect = 0.003, SE = 0.001, 95% CI [0.001, 0.006]). Thus, adolescents with lower levels of parental knowledge may be more likely to involved the deviant peers and IGA. Therefore, pathway from sensation seeking to adolescent IGA was mediated by deviant peer affiliation and moderated by parental knowledge.

## Discussion

In the current study, we tested a moderated mediation model to reveal the relationship between sensation seeking, deviant peer affiliation, parental knowledge and adolescent IGA. First, our results supported our first hypothesis and the social development model (Hawkins and Weis, 1985), as we found that deviant peer affiliation significant mediated the positive association between sensation seeking and adolescent IGA. Sensation seeking can positive predict the affiliation with deviant peer, thus it will lead to higher risk of IGA. Researchers have previously found sensation seeking was positively associated with problem behaviors, and this relationship was mediated by deviant peer affiliation (Hampson et al., 2008). In addition, Li et al. (2016) found that mediation effect of deviant peer affiliation in the association between sensation seeking and problematic Internet use (including overindulgence of games). According to the social development model (Hawkins and Weis, 1985), adolescents integrate into a group of peers and develop behavioral pattern that are similar to those peers. Leung et al. (2016) reported that adolescents increasingly adopt their peers’ behaviors and attitudes. Our current findings support and extend existing field of research which have found that sensation seeking can positive predict adolescent IGA and deviant peer affiliation mediated this relationship.

Second, the moderated mediation model showed that the two stages of the indirect pathways were moderated by parental knowledge. These results supported Hypothesis 2 and the ecological systems theory (Bronfenbrenner, 2005). The findings showed that adolescents with high level of parental knowledge have a lower risk of deviant peer affiliation and IGA than adolescent with low of parental knowledge. According to the ecological systems theory (Bronfenbrenner, 2005), the development of adolescent IGA depends on the interaction of intrapersonal characteristics (e.g., sensation seeking) and environmental factors (e.g., parental knowledge, deviant peer affiliation). In other words, the magnitude of the risk was influence by the effect of sensation seeking on deviant peer affiliation, and the effect of deviant peer affiliation on adolescent IGA depended on the level of parental knowledge about the lives of their adolescents. Specifically, among adolescents with high parental knowledge, parents might provide them good guidance, which, in turn, can help to promote their resilience, reduce the temptation of engaging with deviant peers, and subsequently reduce the likelihood of IGA. Conversely, adolescents with parents who have low knowledge might have more time and opportunities to seek out risk and stimulation (i.e., IGA), integrate into deviant peer groups, and develop IGA through their deviant peer group.

### Limitations and Future Directions

Although the current study contributes to a better understanding of IGA, our results should be interpreted with caution, as this study had several limitations. First, our model explains the relationship between sensation seeking and IGA base on a cross-sectional data; therefore, we cannot test the infer causation. Future studies should adopt a longitudinal design to establish causality and explore other alternative models with multiple time points. Second, our findings may be limited by the method of data collection (e.g., all measures were collected by students’ self-reports). Adolescents may not fully describe variables such as parental knowledge; therefore, further studies could use multiple methods of data collection (e.g., self-reports, parent-reports, teacher-reports). Moreover, all participants were from the southern part of China; future studies should replicate these findings with other cultures as well as other regions of China. Finally, the current study tested the roles of sensation seeking, deviant peer affiliation and parental knowledge among Chinese adolescents’ IGA. However, other factors may also influence the development of IGA during adolescence. Future research should explore the potential roles and mechanism of other factors in the relationship between sensation seeking and adolescent IGA, such as the effect of self-esteem (Corsano et al., 2017), the mechanism of emotional autonomy (Majorano et al., 2015) and attachment relationships (Terrone et al., 2018).

### Implications for Practice

Despite these limitations, these findings have critical implications concerning adolescence development, family education, and psychological health. First, we found that adolescents with elevated levels of sensation seeking were at increased risk for IGA. This relationship suggests that parents and educators should pay greater attention to adolescents with high sensation seeking, as it might contribute to the development of IGA. Second, our results identified deviant peer affiliation as a mediating factor in the relationship between sensation seeking and IGA; therefore, intervention programs could work to reduce the risk of IGA by helping adolescent identify positive and prosocial friendships or assist with developing skills to buffer the influence of deviant peers. Finally, our findings revealed that parental knowledge can moderate the relationship between deviant peer affiliation and IGA. These results demonstrate that adolescents’ IGA might be influenced by their individual characteristics and deviant peer affiliations as well as by familial factor (e.g., parental knowledge). Parents should try to learn more about what adolescents are doing, to help them avoid deviant peer affiliation and effectively intervene to reduce the impact of deviant peers. They may use technology (i.e., smart phone) to keep better track of adolescents (Weisskirch, 2009) or interactive positively (i.e., encouraging adolescent) to more effectively connect with their adolescents (Wang et al., 2012). Thus, parents could provide adolescents with timely guidance about friendships to help reduce the risk of IGA.

## Author Contributions

YT and CY conceived and designed the research, performed the research, and wrote the manuscript. YT, CY, and JL analyzed the data. SL, JL, YL, and WZ revised the article critically for important intellectual content, commented on and approved the final manuscript.

## Conflict of Interest Statement

The authors declare that the research was conducted in the absence of any commercial or financial relationships that could be construed as a potential conflict of interest.
